# Readmissions attributable to skilled nursing facility use after a colectomy: Evidence using propensity scores matching

**DOI:** 10.1371/journal.pone.0215245

**Published:** 2019-04-16

**Authors:** Yubraj Acharya, Amber L. Schilling, Christopher S. Hollenbeak

**Affiliations:** 1 Department of Health Policy and Administration, College of Health and Human Development, The Pennsylvania State University, University Park, Pennsylvania, United States of America; 2 Department of Surgery, Division of Outcomes Research and Quality, The Pennsylvania State University, College of Medicine, Hershey, Pennsylvania, United States of America; Harvard Medical School Teaching Hospital, UNITED STATES

## Abstract

**Background:**

Postacute care (PAC) is a major driver of the rising health care costs in the United States (US). There is limited evidence on the causal effect of skilled nursing facility (SNF) use on readmission after an inpatient colectomy.

**Study design:**

We performed a retrospective analysis of data from the Pennsylvania Health Care Cost Containment Council (PHC4) on 38,635 patients who underwent an inpatient colectomy between 2011 and 2014 in a Pennsylvania hospital. Using propensity scores, we matched patients who were discharged to a SNF to those who were discharged elsewhere. We compared the probability of readmissions within 30 days for the two groups of matched patients in a regression framework. For the subset of patients who were readmitted within 30 days, we assessed whether patients discharged to SNF were readmitted earlier than those discharged to other entities.

**Results:**

The use of a SNF after a colectomy significantly raises the patients’ chance of readmissions within 30 days, even after controlling for their demographic characteristics and illness severity. Based on our estimates, being discharged to a SNF raises the chance of a readmission by 7.7 percentage points. For patients who were admitted within 30 days, we find no association between discharge to a SNF and the timing of readmission.

**Conclusion:**

Sending less severe patients to facilities other than a SNF following inpatient colectomy may help hospitals reduce 30-day readmission rates.

## Introduction

Postacute care (PAC) is a major driver of the rising health care costs in the United States (US) [1,2]. An emerging body of literature has examined the variation in health care spending across the postacute care options—namely, readmissions, and the use of a skilled nursing home facility (SNF), inpatient rehabilitation facility (IRF), outpatient rehabilitation facility (ORF), or a home health agency (HHA) [3,4]. These studies attempt to parse out the contribution of these PAC options to the overall health care spending.

However, generating an accurate estimate of the effect of various PAC alternatives on spending is compounded by two factors. The first factor is selection—the fact that patients who go to a SNF, for example, are inherently different from those who go to an HHA or an IRF. Moreover, the decision to choose one PAC form over another may be determined by the facility’s availability and its proximity to the hospital. In fact, among elderly patients hospitalized for stroke, hip fractures, or lower extremity joint replacement, PAC availability is a strong predictor of PAC use [5]. Buntin et al. find that the farther away the nearest IRF is from the hospital, and the closer the nearest SNF is, the less likely a patient was to go to an IRF [5]. Likewise, if a hospital had a related IRF or SNF, the hospital’s patients were more likely to go there.

The second, related, factor is substitution between the PAC options. A SNF, an HHA, an IRF, and an ORF all differ in terms of the intensity, type of care they provide, and how they are reimbursed (when applicable), thus catering to different type of patients. Nonetheless, the hospital where a patient undergoes the surgery has some flexibility in deciding where the patient should go and when he/she should be discharged. Some of the recent policy measures, such as the Hospital Readmissions Reduction Program (HRRP), may encourage the hospitals to utilize this flexibility to engage in “gaming.” For example, in an effort to keep readmissions low, the hospitals may choose to send less severe patients to a PAC facility with more intensive care, such as a SNF or an IRF, than they would have in the absence of HRRP. Such behavior may reduce readmissions but raise the utilization of certain types of PAC facilities, thus limiting the net change in the overall health care spending.

In an attempt to understand the effect of one PAC option (SNF utilization) on readmissions, we asked: by how much does the risk of a readmission change if a patient is discharged to a SNF instead of one of the other PAC facilities? In answering this question, we estimated how much switching takes place between PAC options, mainly SNF use and readmissions, and, more importantly, the causal effect of SNF use on readmissions. To address the two challenges discussed in the previous paragraphs, we used the propensity score matching (PSM) technique used by Garrido et al. (2014) [6].

Our contribution to the literature is primarily methodological. A number of prior studies have examined the association between SNF use (or PAC use generally) and readmissions following a major surgery, with mixed evidence. For example, Chen et al. (2012) found no association between SNF rate and 30-day readmission rates for patients with heart failure or acute myocardial infarction (AMI) [7]. Conversely, Keswani et al. (2016) have found that lower extremity joint replacement patients discharged to SNFs had higher readmission rates than those discharged elsewhere [8]. These studies are vulnerable to the limitations discussed in the previous two paragraphs.

To our knowledge, Chen et al. (2018) is the only study that has attempted to causally link SNF use to readmissions [9]. The authors used Medicare claims data for patients who underwent a total hip replacement (THR) or coronary artery bypass grafting (CABG) between 2008 and 2013. They accounted for selection by assessing the relationship between the change in readmissions and the change in SNF use between the two periods at the hospital level. The present study extends on that work both methodologically (using PSM), as well as by using a data set that is more representative of the general population. Specifically, Chen et al. use fee-for-service Medicare claims data for older Americans, so results may not generalize to other populations, such as younger patients and patients with private insurance or Medicaid. Our study overcomes this limitation by using a sample of patients from an all-payer administrative discharge database. We are also able to account for the severity of the patients’ illness more accurately, and hence reduce selection bias, as our data set contains details on the approach and the urgency of the surgery.

## Materials and methods

### Data sources

This was an observational study utilizing data from the Pennsylvania Health Care Cost Containment Council (PHC4) database for the period between January 2011 to December 2014. PHC4 is a state-run agency whose goal is to help contain the rapidly-increasing health care costs and to make healthcare provider performance information more readily available to the public. Each year, PHC4 collects discharge records from over 4 million individuals in inpatient, ambulatory, and freestanding surgery centers located throughout the state of Pennsylvania. De-identified patient demographic information, diagnostic and procedural codes, hospital information, and financial data are available within PHC4.

An ethics approval was not required for this study as it used de-identified, secondary data.

### Population studied

We restricted the sample to adult patients (≥18 years) in Pennsylvania who underwent an inpatient colectomy between January 2011 and December 2014. The *International Classification of Disease*, *9*^*th*^ *Revision*, *Clinical Modification* (ICD-9-CM) procedure codes we used in order to identify the colectomy cases are in S1 Table. To clarify, all six procedural codes available within PHC4 (one primary and five secondary) were searched in identifying cases for this study, and all surgical urgencies were included (emergent, urgent, and elective).

### Outcome measures

Our primary outcome of interest was a binary variable indicating whether a patient was readmitted for any cause within 30 days of discharge from the hospital following a colectomy. As PHC4 data does not indicate whether the readmission was planned or unplanned, our readmission variable included all types of readmissions that occurred to any hospital within Pennsylvania.

A secondary outcome of interest included elapsed time (in days) between discharge from the hospital following the index admission and readmission. PHC4 tracks the number of days from a patient’s discharge date until their next admission to any hospital in Pennsylvania.

### Exposure variable

Our key exposure variable was a binary variable indicating whether a patient was discharged to a SNF following a colectomy. PHC4 contains information on whether the patient was discharged to a SNF, home (self-care), to an organized home health organization.

### Confounders

Patient-level characteristics hypothesized to influence the use of a SNF (as well as the risk of readmission) were age, race, sex, surgical approach (laparoscopic vs non-laparoscopic), surgical urgency (emergent, urgent, or elective), primary indication for the colectomy, ostomy use, surgical urgency, transfer status, primary payer, year of surgery, length of stay (LOS) for the index admission, and comorbidities. All of these were included in our regression analyses and the PSM algorithm.

Following Buntin et al. (2005), we classified patients into age quartiles to account for nonlinear effects of age on SNF use and readmissions [5]. Surgical approach, surgical urgency, Charlson Comorbidity Index, and LOS were all utilized as surrogate markers for patient illness severity. We considered the ICD-9-CM procedure codes 458.1, 173.1–173.6, and 173.9 as using the laparoscopic surgical approach. Colectomy cases not otherwise identified as laparoscopic were assumed to have been performed using an open approach.

The Charlson Comorbidity Index reflects a patient’s risk of death and is based on the presence of 17 comorbid conditions (myocardial infarction, congestive heart failure, peripheral vascular disease, cerebrovascular disease, dementia, chronic pulmonary disease, rheumatologic disease, peptic ulcer disease, mild liver disease, moderate / severe liver disease, diabetes, diabetes with chronic complications, hemiplegia / paraplegia, renal disease, any malignancy, metastatic solid tumors, and AIDS). Following Deyo et al., the index weighs each condition according to relative severity and likelihood of contributing to death [10]. Higher overall index implies higher mortality risk.

To account for factors at the hospital level, we included number of colectomy admissions per year and the hospital’s geographic region. Hospital geographical location is coded within PCH4 as one of nine regions (see S2 Table).

### Statistical analysis

Observations with missing information on 30-day readmission (n = 4,444) and demographic data required for matching (n = 1,542) were dropped from the analysis. In our univariate analyses, characteristics of patients discharged to a SNF versus those discharged elsewhere were compared using Student’s t-tests for continuous variables and chi-square tests for binary and categorical variables.

In the PSM analysis, each patient who was discharged to a SNF was matched to an otherwise similar patient in the sample (but who was discharged elsewhere) based on all aforementioned covariates. Matching was performed 1:1, without replacement, with a *k*-nearest neighbor approach, and a caliper restriction of 0.2 standard deviations.

We utilized logistic regression to estimate the relationship between readmissions and SNF use for the matched sample while accounting for the potential confounders described previously. The average treatment effect was calculated and is reported as the average effect of treatment on the treated (ATT) value. For this study, the ATT value is interpreted as the percent difference in likelihood of being readmitted if a patient is discharged to a SNF (taken as the “treatment effect”) versus if they are discharged elsewhere. To interpret the estimates causally, we assumed that a patient who was discharged to a SNF would have had the same probability of being readmitted as his/her matched counterpart who was discharged elsewhere if the former had also been discharged elsewhere. Following this assumption, the only difference in the probability of readmission would thereby be due to the first patient being discharged to a SNF. To test the robustness of our model assumptions, we also performed the analysis using a 1:2 matching algorithm (matching each patient discharged to a SNF to two patients who were discharged elsewhere).

Finally, we estimated the relationship between the days-to-readmission and SNF use using linear regression on the matched sample of patients. We performed all statistical analysis using Stata version 12.1 (Stata Corp, College Station, TX).

## Results

In our sample of 38,635 patients who underwent a colectomy in Pennsylvania during 2011–2014, the average age was 63 years (Table 1, Column 1). Eighty-eight percent (88.4%) of these individuals were White and approximately 46% were male. Most colectomies were performed electively and using a non-laparoscopic approach (61.1% and 63.1%, respectively). The most common indications for having the colectomy performed were cancer (29.1%) and diverticular disease (25.1%); the “other” indication category in Table 1 includes unspecified septicemia, intestinal obstruction, non-infectious enteritis, intestinal perforation, intestinovesical fistula. Most patients (97.7%) did not have a diagnosis indicating ostomy use (colostomy or ileostomy). Of the total number of patients, 18,931 (49%) were on Medicare, 15,840 (41%) had private insurance, and 3,090 (8%) were on Medicaid.

**Table 1:** Demographic characteristics and selected outcomes for the colectomy cohort prior to matching, stratified by discharge to SNF.

	Entire Colectomy	Discharged	Not Discharged	
	Cohort	to SNF	to SNF	
Variable	*(N = 38*,*635)*	*(N = 5*,*540)*	*(N = 33*,*095)*	P-Value
Age (mean, yrs)	63.0	76.9	60.7	<0.0001
18–54, %	28.0	4.4	31.9	
55–64, %	23.4	9.2	25.8	
65–74, %	23.6	21.3	24.0	
≥75, %	24.9	65.0	18.2	
Race				<0.0001
White, %	88.4	88.2	88.5	
Black, %	8.2	9.4	8.0	
Other, %	3.4	2.4	3.5	
Sex				<0.0001
Male, %	45.9	35.0	47.7	
Female, %	54.1	65.0	52.3	
Surgical Approach				<0.0001
Laparoscopic, %	36.9	15.4	40.5	
Non-Laparoscopic, %	63.1	84.6	59.5	
Primary Indication				<0.0001
Diverticular Disease, %	25.1	15.4	26.7	
Cancer, %	29.1	33.7	28.3	
Other, %	45.9	50.9	45.0	
Ostomy				<0.0001
Yes, %	2.3	4.7	1.9	
No, %	97.7	95.3	98.1	
Surgical Urgency				<0.0001
Emergent, %	29.9	59.7	24.9	
Urgent, %	9.1	11.3	8.7	
Elective, %	61.1	29.0	66.4	
Transfer, %	2.0	6.7	1.2	<0.0001
Payer				<0.0001
Medicare, %	48.8	85.9	42.5	
Medicaid, %	7.7	4.3	8.3	
Other Gov't Payer, %	0.7	0.3	0.8	
Commercial, %	41.4	9.0	46.8	
Self-Paying, %	1.0	0.3	1.1	
Other/Unknown, %	0.5	0.2	0.5	
Charlson Comorbidity Index Score (mean)	2.2	3.2	2.0	<0.0001
0, %	39.0	18.5	42.4	
1, %	14.9	15.4	14.8	
≥2, %	46.2	66.1	42.8	
Discharge Destination				n/a
SNF, %	14.3	100.0	0.0	
Home, %	57.6	0.0	67.3	
Home Health, %	27.2	0.0	31.7	
Other, %	0.9	0.0	1.0	
Region of Pennsylvania				<0.0001
Northwest, %	6.2	7.3	6.1	
Southwest, %	28.7	29.0	28.6	
North Central, %	5.1	4.3	5.3	
South Central, %	15.1	10.8	15.8	
Northeast, %	6.7	6.5	6.7	
Southeast, %	38.2	42.2	37.5	
Hospital Volume (mean no. of admissions per year)	616.5	567.5	624.7	<0.0001
≤270, %	25.0	28.9	24.3	
271–470, %	23.3	24.7	23.1	
471–800, %	23.6	23.6	23.6	
>800, %	28.1	22.8	29.0	
Year				0.975
2011, %	25.6	25.3	25.6	
2012, %	25.1	24.9	25.1	
2013, %	24.8	25.0	24.8	
2014, %	24.5	24.8	24.5	
Length of Stay (mean, days)	8.3	15.1	7.2	<0.0001
0–4, %	28.2	2.9	32.4	
5–6, %	23.8	8.5	26.3	
7–10, %	25.2	27.4	24.8	
>11, %	22.9	61.2	16.5	
Readmission w/in 30-days	13.5	25.1	11.6	<0.0001
Mean days to readmission*	10.7	11.5	10.4	0.0001
0–4, %	4.0	6.7	3.6	
5–8, %	2.8	5.0	2.4	
9–15, %	3.1	5.8	2.6	
16–30, %	3.7	7.7	3.0	

This table shows the characteristics of the patients in our sample, stratified by their post-discharge destination (SNF versus elsewhere), before those discharged to SNF were matched to those discharged to another destination.

*Among those who were readmitted within 30 days (n = 5257 for overall; n = 1396 for SNF; n = 3896 for non-SNF).

Overall, patients in this colectomy cohort stayed in the hospital for an average of 8.3 days, and 13.5% were readmitted to the hospital within 30-days. As shown in S3 Table, the most common causes for readmission were post-operative infection (14.4% of those readmitted), digestive system complication (10.0%), acute renal failure (5.0%), septicemia (3.8%), and intestinal obstruction (3.2%). Dehydration (a common cause of readmission following colectomy) was the primary diagnosis for the readmission in 2.0% of all readmissions. The majority of these patients (nearly 58%) returned home (self-care) following their colectomy, while 14% went to a SNF and 27% went to HHA.

When patients are compared according to discharge destination (SNF or non-SNF), SNF patients differed significantly from those who were discharged to another destination on all of the characteristics included in Table 1 (all p-values <0.0001), with the exception of year that the colectomy was performed. Notably, patients discharged to a SNF were significantly older (76.9 versus 60.7 years old; p<0.0001), had higher proportions of females (65.0% versus 52.3%; p<0.0001), and were more likely to have had an open as opposed to a laparoscopic surgery (84.6% versus 59.5% for open and 15.4% versus 40.5% for laparoscopic, respectively; p<0.0001). SNF patients had slightly different distributions of primary indications and ostomy use compared to non-SNF patients (p<0.0001 for both). SNF patients were almost twice as likely to be covered by Medicare compared to those who were discharged elsewhere (85.9% versus 42.5%; p<0.0001), had slightly higher Charlson Comorbidity Index Scores (3.2 versus 2.0; p<0.0001), and stayed twice as long in the hospital during the index admission (15.1 days versus 7.2 days; p<0.0001).

Table 2 draws comparisons among the matched cohort (patients discharged to a SNF matched to non-SNF patients). Patients in the matched groups did not differ significantly in distributions of race, sex, surgical approach, primary indication, ostomy use, surgical urgency, transfer status, primary payer mix, Charlson Comorbidity Index, geographic region, mean number of admissions in their hospital, and year of surgery. Although age remained statistically significant, the substantive difference was small (1.6 years, p<0.0001). Length of stay was also significantly different in the matched cohort (14.4 days for SNF and 11.9 days for non-SNF; p<0.0001).

**Table 2:** Demographic characteristics for the matched colectomy cohort, stratified by discharge to SNF (matched 1:1).

	Discharged	Not Discharged	
	to SNF	to SNF	
Variable	*(N = 4484)*	*(N = 4484)*	P-Value
Age (mean, yrs)	75.5	73.9	<0.0001
18–54, %	5.5	4.6	
55–64, %	11.3	11.0	
65–74, %	25.6	25.8	
≥75, %	57.6	58.7	
Race			0.925
White, %	88.4	88.3	
Black, %	9.1	9.0	
Other, %	2.6	2.7	
Sex			0.051
Male, %	39.3	37.3	
Female, %	60.7	62.7	
Surgical Approach			0.913
Laparoscopic, %	18.4	18.3	
Non-Laparoscopic, %	81.6	81.7	
Primary Indication			0.245
Diverticular Disease, %	16.1	14.9	
Cancer, %	36.0	37.1	
Other, %	47.9	47.9	
Ostomy			0.301
Yes, %	4.1	3.7	
No, %	95.9	96.3	
Surgical Urgency			0.702
Emergent, %	52.6	52.5	
Urgent, %	12.2	11.7	
Elective, %	35.2	35.8	
Transfer, %	3.8	4.0	0.664
Payer			0.390
Medicare, %	82.9	84.1	
Medicaid, %	5.2	5.1	
Other Gov't Payer, %	0.4	0.5	
Commercial, %	11.0	9.7	
Self-Paying, %	0.4	0.3	
Other/Unknown, %	0.2	0.2	
Charlson Comorbidity Index Score (mean)	3.1	3.2	0.299
0, %	20.6	19.6	
1, %	15.3	14.4	
≥2, %	64.1	66.0	
Region of Pennsylvania			0.892
Northwest, %	7.4	7.6	
Southwest, %	29.8	29.8	
North Central, %	4.7	4.9	
South Central, %	12.1	11.4	
Northeast, %	6.9	6.7	
Southeast, %	39.1	39.6	
Hospital Volume (mean no. of admissions per year)	573.8	563.2	0.269
≤270, %	28.2	28.0	
271–470, %	25.0	25.4	
471–800, %	23.3	23.8	
>800, %	23.6	22.9	
Year			0.925
2011, %	25.5	25.3	
2012, %	24.9	24.4	
2013, %	25.0	25.6	
2014, %	24.6	24.7	
Length of Stay (mean, days)	14.4	11.9	<0.0001
0–4, %	3.6	3.2	
5–6, %	10.4	10.5	
7–10, %	33.1	33.7	
>11, %	52.9	52.5	

This table shows the characteristics of the patients in our sample, stratified by their post-discharge destination (SNF versus elsewhere), after those discharged to SNF were matched to those discharged to another destination on the set of characteristics included in this table.

Table 3 presents how our primary and secondary outcomes differed in the matched cohort analysis. Patients discharged to a SNF had nearly 8% higher likelihood of being readmitted to the hospital within 30 days of initial discharge (24.2% versus 16.5% with Average Effect of Treatment on the Treated [ATT] as 7.7%; p<0.0001). The percent difference in readmission rate remained statistically significant even after controlling for potential confounders through the matching process. Although the difference decreased from 13.5 percentage points a 7.7 percentage points (decreasing from 25.1% to 24.2% among SNF patients and increasing from 11.6% to 16.5% among non-SNF patients), it remained statistically significant at the 1% significance level. Even if we consider the lowest bound of the average treatment effect reported in the table, a patient’s readmission rate increased by 6.0 percentage points if he/she was discharged to a SNF (95% CI: 6.0% to 9.4%). The full set of results from a logistic regression of readmissions on SNF use and the covariates for the matched sample, with odds ratios reported for all covariates, are in S4 Table.

**Table 3:** Results of a propensity score matching analysis for 30-day readmission (discharge to SNF treated as the treatment effect; 1:1 matching).

	Discharged	Not Discharged		95% Confidence		
Outcome	to SNF	to SNF	ATT	*Lower*	*Upper*	P-value
30-day Readmission	24.22%	16.50%	7.72%	6.03%	9.40%	<0.0001
Mean days to readmission*	11.47	11.51	-0.04	-0.87	0.79	0.918

ATT, average effect of treatment on the treated. Patients were matched on all covariates included in Table 1.

*For those readmitted within 30 days.

**Fig 1:**
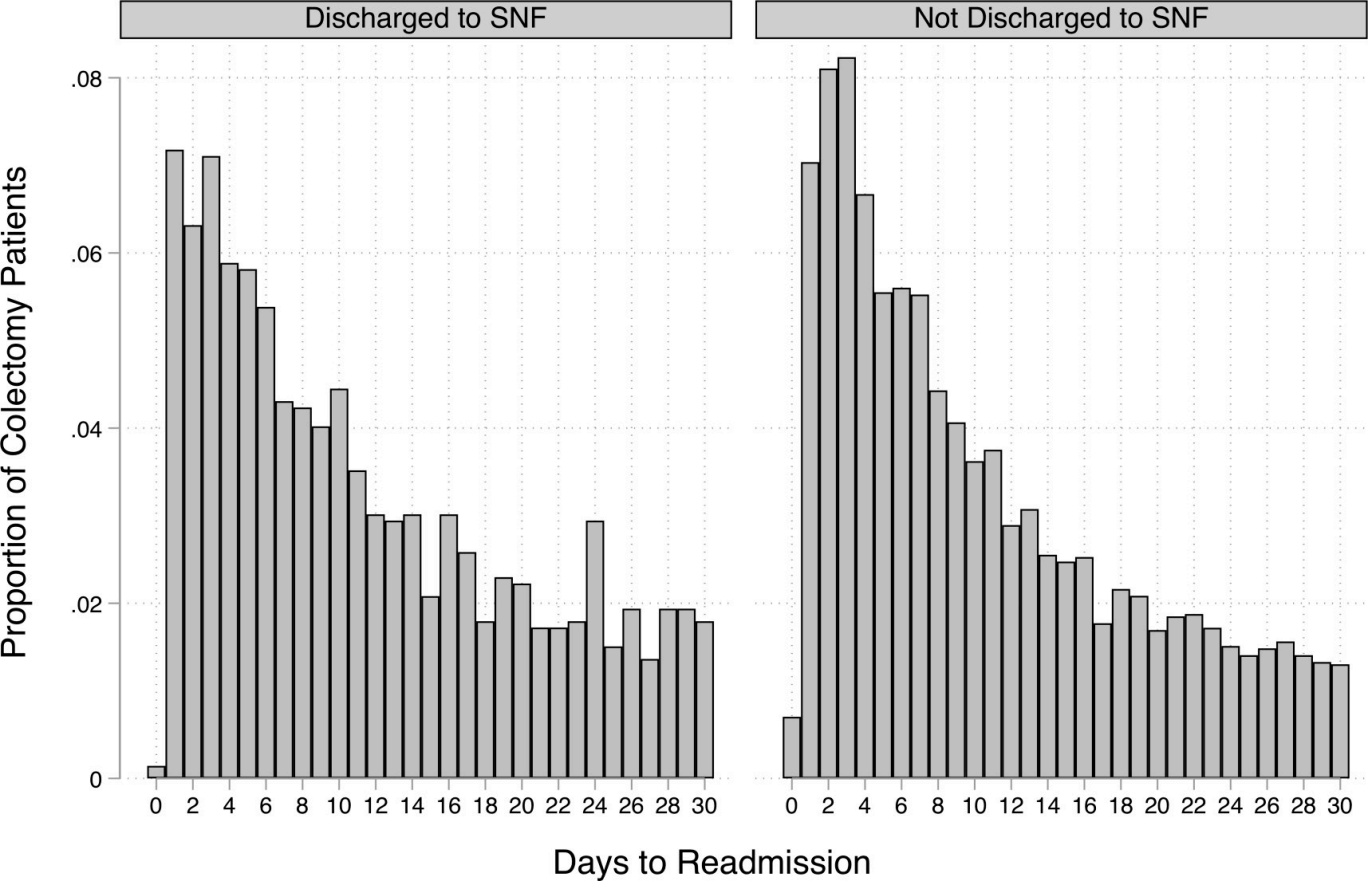
Days to readmission for those readmitted within 30 days, stratified by discharge to SNF.

We found no effect of being discharged to a SNF on the timing of readmission, conditional on being readmitted within 30 days. The proportion of patients who were readmitted on each of the days between day 0 and day 30 after discharge is shown in Fig 1, separately for those discharged to a SNF and those discharged elsewhere. Both types of patients tended to be readmitted early on (days 0–5). After matching, and controlling for covariates, the mean days to readmission was close to 11.5 days for patients discharged to a SNF as well as those discharged elsewhere (Table 3). The full set of results from the linear regression of days to readmission on SNF use and covariates for the matched sample, with coefficients reported for all covariates, are in S5 Table.

Our main result—that 7.7% of the readmissions following colectomy in our sample were attributable to the use of SNF—was robust to alternative matching methods. In S6 Table, we replicated Table 3, but for the sample with one patient discharged to SNF matched with two patients discharged elsewhere. The table shows that being discharged to a SNF raised the readmission rate by approximately 8.6 percentage points (p<0.0001). We also re-estimated the relationship between days-to-admission and SNF utilization using a generalized linear model (with gamma family distribution and log-link function). Consistent with finding from the OLS, SNF use was not associated with days-to-readmission (S7 Table).

## Discussion

In an attempt to generate a causal estimate of the effect of SNF utilization on readmissions, we used the PSM technique that is acceptable but, in our view, underused in health services research. We also used all-payer data, thus addressing the external validity concerns common to prior studies that use Medicare-only data. In our analysis, we were able to control for a wider range of potential confounders, especially those related to the patient’s illness severity (e.g., the urgency of the surgery and the approach used), than has been done previously. We found that being discharged to a SNF was significantly associated with a patient’s chance of readmission within 30 days following colectomy, even after controlling for demographic characteristics and surrogate markers for illness severity. Based on our estimates, being discharged to a SNF raises the risk of readmission by 7.7 percentage points. This is a large effect, given that the overall readmissions rate in our sample is 13.5%.

Previous studies have shown that spending patterns on postacute care vary by the type of postacute care settings [3]. For example, Chen et al. (2012) have found that the choice of postacute care setting explains a substantial amount of variation in postacute care spending for fee-for-service Medicare beneficiaries who have undergone total hip replacement, coronary artery bypass grafting or colectomy [4]. For colectomy, the authors find that the variation on postacute care spending is “equally explained by the choice of inpatient rehabilitation facilities, the choice of skilled nursing facilities and skilled nursing facility intensity” (p. 89). Our findings suggest that the choice of skilled nursing facilities raises postacute care spending also indirectly by raising the risks of readmissions substantially.

Our findings should be understood in light of a number of related limitations. First, although we have used a method that helps us reduce confounding due to health and other characteristics associated with disposition after colectomy as well as the likelihood of subsequent re-admission, we cannot still claim that the estimates are fully causal. A natural next step in this line of research is to match patients on additional confounders, primarily the availability of a SNF, additional measures of patient severity, and post-operative intra-hospital complications, in order to further confirm that the estimated effect is not due to those confounders. We were unable to do so in this study because of the lack of such information in the PHC4 data. Second, it would have been more appropriate to examine the chances of *unplanned* admissions as the outcome. Unfortunately, the data set does not contain information on whether the admission was unplanned. Third, from a policy point of view, it is still unclear what ultimately drives the observed effect, especially after we have accounted for selection bias in SNF utilization due to wide range of factors. Finally, we are unable to confirm the external validity of our findings to the rest of the US population. Future research could examine whether the large effect we found holds true in other settings.

Despite these limitations and unanswered questions, the key implication of our finding is clear: sending less severe patients to facilities other than a SNF following inpatient colectomy may help hospitals reduce 30-day readmission rates and reduce postacute care costs. This finding is consistent with previous cross-sectional studies and is poignant in light of HRRP, as many hospitals are already implementing a variety of care interventions to reduce readmissions, such as arranging early discharge follow-up, reconciling medications, performing follow-up phone calls, and partnering with other local hospitals or care facilities after a major surgery [11].

## Supporting information

S1 Table ICD-9-CM procedure codes utilized to identify the colectomy cohort. (DOCX)

S2 Table Geographical region codes available in PHC4. (DOCX)

S3 Table Top causes of readmission following colectomy, based on the principal diagnosis listed for readmissions. (DOCX)

S4 Table Results from the logistic regression of 30-day readmission on SNF utilization for the matched cohort of patients. AROC = 0.6283. Sample Size for Matched Cohort = 8968.
(DOCX)

S5 Table Results from the regression of days to readmission on SNF utilization for the matched cohort of patients. R-squared = 0.0145. Sample Size for Matched Cohort = 1890.
(DOCX)

S6 Table Results of a propensity score matching analysis for 30-day readmission (discharge to SNF treated as the treatment effect; 2:1 matching). ATT: average effect of treatment on the treated. Notes: 1) Patients were matched on all covariates included in Table 1. 2) This table shows the effect of being discharged to a SNF on readmissions rate, after the patients were matched on all covariates included in Table 1. Each patient discharged to a SNF was matched to two patients who were discharged to another destination. 3) Full set of regression results similar to those in S1 Table and S2 Table are available from the authors on request.
(DOCX)

S7 Table Marginal effects from a generalized linear model regression of days to readmissions on SNF utilization on the matched cohort of patients. (DOCX)
