# Supplementary material for: Readmissions attributable to skilled nursing facility use after a colectomy: Evidence using propensity scores matching
Source: PLoS One. 2019 Apr 16;14(4):e0215245. doi: 10.1371/journal.pone.0215245 (PMC6467448; doi:10.1371/journal.pone.0215245)
Supplement: S1 Table — (DOCX) [file pone.0215245.s001.docx]

S1 Table. ICD-9-CM procedure codes utilized to identify the colectomy cohort.

| **ICD-9-CM Code** | **Definition** |
| --- | --- |
|  |  |
| 45.71 | Open and other multiple segmental resection of large intestine |
| 45.72 | Open and other cecectomy |
| 45.73 | Open and other right hemicolectomy |
| 45.74 | Open and other resection of transverse colon |
| 45.75 | Open and other left hemicolectomy |
| 45.76 | Open and other sigmoidectomy |
| 45.79 | Other and unspecified partial excision of large intestine |
| 45.81 | Laparoscopic total intra-abdominal colectomy |
| 45.82 | Open total intra-abdominal colectomy |
| 17.31 | Laparoscopic multiple segmental resection of large intestine |
| 17.32 | Laparoscopic cecectomy |
| 17.33 | Laparoscopic right hemicolectomy |
| 17.34 | Laparoscopic resection of transverse colon |
| 17.35 | Laparoscopic left hemicolectomy |
| 17.36 | Laparoscopic sigmoidectomy |
| 17.39 | Other laparoscopic partial excision of large intestine. |
|  |  |
