# Supplementary material for: Readmissions attributable to skilled nursing facility use after a colectomy: Evidence using propensity scores matching
Source: PLoS One. 2019 Apr 16;14(4):e0215245. doi: 10.1371/journal.pone.0215245 (PMC6467448; doi:10.1371/journal.pone.0215245)
Supplement: S2 Table — (DOCX) [file pone.0215245.s002.docx]

S2 Table. Geographical region codes available in PHC4.

| **Hospital Region Code** | **Geographical Region** |
| --- | --- |
|  |  |
| 1 | South West |
| 2 | North West |
| 3 | South West |
| 4 | North Central |
| 5 | South Central |
| 6 | North East |
| 7 | South East |
| 8 | South East |
| 9 | South East |
|  |  |
