# Supplementary material for: Readmissions attributable to skilled nursing facility use after a colectomy: Evidence using propensity scores matching
Source: PLoS One. 2019 Apr 16;14(4):e0215245. doi: 10.1371/journal.pone.0215245 (PMC6467448; doi:10.1371/journal.pone.0215245)
Supplement: S3 Table — (DOCX) [file pone.0215245.s003.docx]

S3 Table. Top causes of readmission following colectomy, based on the principal diagnosis listed for readmissions.

| ICD-9 Principal Diagnosis Code for Readmission Hospitalization | Definition | Frequency | Percentage of all 30-day Readmissions |
| --- | --- | --- | --- |
|  |  |  |  |
| 998.59 | Postoperative Infection | 786 | 14.4% |
| 997.49, 997.4 | Digestive System Complication | 548 | 10.0% |
| 584.9 | Acute Renal Failure / Acute Kidney Injury (non-traumatic) | 271 | 5.0% |
| 38.9 | Septicemia | 207 | 3.8% |
| 560.9 | Intestinal Obstruction | 175 | 3.2% |
| 8.45 | C.diff Infection (Pseudomembranous colitis) | 116 | 2.1% |
| 276.51 | Dehydration | 111 | 2.0% |
| 415.19 | Pulmonary Embolism | 100 | 1.8% |
| 599.0 | Urinary Tract Infection | 85 | 1.6% |
| 567.22 | Peritoneal Abscess | 76 | 1.4% |
|  |  |  |  |
