# Supplementary material for: Readmissions attributable to skilled nursing facility use after a colectomy: Evidence using propensity scores matching
Source: PLoS One. 2019 Apr 16;14(4):e0215245. doi: 10.1371/journal.pone.0215245 (PMC6467448; doi:10.1371/journal.pone.0215245)
Supplement: S4 Table — AROC = 0.6283. Sample Size for Matched Cohort = 8968. (DOCX) [file pone.0215245.s004.docx]

S4 Table. Results from the logistic regression of 30-day readmission on SNF utilization for the matched cohort of patients.

|  | **Odds** | **95% Confidence** | |  |
| --- | --- | --- | --- | --- |
| **Covariate** | **Ratio** | *Lower* | *Upper* | **P-value** |
|  |  |  |  |  |
| Age (yrs) |  |  |  |  |
| 18-54 | 1.22 | 0.92 | 1.60 | 0.16 |
| 55-64 | 1.22 | 1.00 | 1.48 | 0.06 |
| 65-74 | 1.11 | 0.97 | 1.26 | 0.13 |
| ≥75 | REFERENCE |  |  |  |
|  |  |  |  |  |
| Race |  |  |  |  |
| White | REFERENCE |  |  |  |
| Black | 1.20 | 1.00 | 1.44 | 0.05 |
| Other | 1.34 | 0.99 | 1.82 | 0.06 |
|  |  |  |  |  |
| Sex |  |  |  |  |
| Male | 1.01 | 0.90 | 1.12 | 0.89 |
| Female | REFERENCE |  |  |  |
|  |  |  |  |  |
| Surgical Approach | |  |  |  |
| Laparoscopic | 0.77 | 0.66 | 0.89 | 0.00 |
| Non-Laparoscopic | REFERENCE |  |  |  |
|  |  |  |  |  |
| Primary Indication | |  |  |  |
| Diverticular Disease, % | REFERENCE |  |  |  |
| Cancer, % | 0.94 | 0.77 | 1.14 | 0.51 |
| Other, % | 1.48 | 1.26 | 1.73 | <0.0001 |
|  |  |  |  |  |
| Ostomy |  |  |  |  |
| Yes, % | 1.30 | 1.01 | 1.67 | 0.04 |
| No, % | REFERENCE |  |  |  |
|  |  |  |  |  |
| Surgical Urgency | |  |  |  |
| Emergent | 0.98 | 0.86 | 1.11 | 0.74 |
| Urgent | 0.94 | 0.78 | 1.13 | 0.54 |
| Elective | REFERENCE |  |  |  |
|  |  |  |  |  |
| Transfer | 1.19 | 0.91 | 1.55 | 0.20 |
|  |  |  |  |  |
| Payer |  |  |  |  |
| Medicare | REFERENCE |  |  |  |
| Medicaid | 0.79 | 0.60 | 1.04 | 0.10 |
| Other Gov't Payer | 1.10 | 0.52 | 2.31 | 0.81 |
| Commercial | 0.91 | 0.75 | 1.10 | 0.34 |
| Self-Paying | 0.71 | 0.29 | 1.76 | 0.46 |
| Other/Unknown | 0.18 | 0.02 | 1.34 | 0.09 |
|  |  |  |  |  |
| Charlson Comorbidity Index Score | | |  |  |
| 0 | 0.76 | 0.65 | 0.88 | <0.0001 |
| 1 | 0.87 | 0.74 | 1.03 | 0.10 |
| ≥2 | REFERENCE |  |  |  |
|  |  |  |  |  |
| Region of Pennsylvania | |  |  |  |
| Northwest | 0.84 | 0.67 | 1.05 | 0.12 |
| Southwest | 0.98 | 0.86 | 1.11 | 0.71 |
| North Central | 1.04 | 0.80 | 1.35 | 0.76 |
| South Central | 0.77 | 0.63 | 0.93 | 0.01 |
| Northeast | 0.98 | 0.79 | 1.22 | 0.87 |
| Southeast | REFERENCE |  |  |  |
|  |  |  |  |  |
| Hospital Volume (mean no. of admissions per year) | | | |  |
| ≥270 | 1.00 | 0.86 | 1.18 | 0.95 |
| 271-470 | 0.86 | 0.73 | 1.01 | 0.06 |
| 471-800 | 1.01 | 0.86 | 1.18 | 0.94 |
| >800 | REFERENCE |  |  |  |
|  |  |  |  |  |
| Year |  |  |  |  |
| 2011 | REFERENCE |  |  |  |
| 2012 | 0.93 | 0.80 | 1.08 | 0.32 |
| 2013 | 0.95 | 0.82 | 1.10 | 0.51 |
| 2014 | 0.91 | 0.78 | 1.06 | 0.22 |
|  |  |  |  |  |
| Length of Stay (mean, days) | | |  |  |
| 0-4 | REFERENCE |  |  |  |
| 5-6 | 1.46 | 0.99 | 2.17 | 0.06 |
| 7-10 | 1.36 | 0.94 | 1.97 | 0.11 |
| >11 | 2.08 | 1.44 | 3.02 | <0.0001 |
|  |  |  |  |  |
| Discharged to SNF | 1.64 | 1.47 | 1.82 | <0.0001 |
|  |  |  |  |  |
|  | |  | | |

*AROC=0.6283. Sample Size for Matched Cohort = 8968.*
