# Supplementary material for: Readmissions attributable to skilled nursing facility use after a colectomy: Evidence using propensity scores matching
Source: PLoS One. 2019 Apr 16;14(4):e0215245. doi: 10.1371/journal.pone.0215245 (PMC6467448; doi:10.1371/journal.pone.0215245)
Supplement: S5 Table — R-squared = 0.0145. Sample Size for Matched Cohort = 1890. (DOCX) [file pone.0215245.s005.docx]

S5 Table. Results from the regression of days to readmission on SNF utilization for the matched cohort of patients.

|  |  | **95% Confidence** | | |  |
| --- | --- | --- | --- | --- | --- |
| **Covariate** | **Coefficient** | *Lower* | *Upper* | | **P-value** |
|  |  |  |  | |  |
| Age (yrs) |  |  |  | |  |
| 18-54 | 1.13 | -0.74 | 2.99 | | 0.24 |
| 55-64 | 0.32 | -1.04 | 1.67 | | 0.65 |
| 65-74 | 0.14 | -0.81 | 1.09 | | 0.78 |
| ≥75 | REFERENCE |  |  | |  |
|  |  |  |  | |  |
| Race |  |  |  | |  |
| White | REFERENCE |  |  | |  |
| Black | -0.41 | -1.68 | 0.85 | | 0.52 |
| Other | 0.19 | -2.08 | 2.47 | | 0.87 |
|  |  |  |  | |  |
| Sex |  |  |  | |  |
| Male | -0.38 | -1.16 | 0.39 | | 0.33 |
| Female | REFERENCE |  |  | |  |
|  |  |  |  | |  |
| Surgical Approach | |  |  | |  |
| Laparoscopic | -0.11 | -1.23 | 1.00 | | 0.84 |
| Non-Laparoscopic | REFERENCE |  |  | |  |
|  |  |  |  | |  |
| Primary Indication | |  |  | |  |
| Diverticular Disease, % | REFERENCE |  |  | |  |
| Cancer, % | -0.89 | -2.27 | 0.50 | | 0.21 |
| Other, % | -0.86 | -2.03 | 0.30 | | 0.15 |
|  |  |  |  | |  |
| Ostomy |  |  |  | |  |
| Yes, % | 0.61 | -1.26 | 2.49 | | 0.52 |
| No, % | REFERENCE |  |  | |  |
|  |  |  |  | |  |
| Surgical Urgency | |  |  | |  |
| Emergent | -0.24 | -1.12 | 0.65 | | 0.60 |
| Urgent | 0.68 | -0.62 | 1.99 | | 0.31 |
| Elective | REFERENCE |  |  | |  |
|  |  |  |  | |  |
| Transfer | -0.54 | -2.52 | 1.43 | | 0.59 |
|  |  |  |  | |  |
| Payer |  |  |  | |  |
| Medicare | REFERENCE |  |  | |  |
| Medicaid | 0.47 | -1.38 | 2.32 | | 0.62 |
| Other Gov't Payer | -1.03 | -6.56 | 4.50 | | 0.72 |
| Commercial | -0.18 | -1.45 | 1.09 | | 0.78 |
| Self-Paying | 0.38 | -5.21 | 5.97 | | 0.90 |
| Other/Unknown | n/a |  |  | |  |
|  |  |  |  | |  |
| Charlson Comorbidity Index Score | | |  | |  |
| 0 | -0.23 | -1.32 | 0.86 | | 0.68 |
| 1 | -0.15 | -1.30 | 1.01 | | 0.81 |
| ≥2 | REFERENCE |  |  | |  |
|  |  |  |  | |  |
| Region of Pennsylvania | |  |  | |  |
| Northwest | 0.94 | -0.71 | 2.60 | | 0.26 |
| Southwest | 0.25 | -0.70 | 1.19 | | 0.61 |
| North Central | 0.55 | -1.32 | 2.42 | | 0.56 |
| South Central | 1.14 | -0.24 | 2.52 | | 0.11 |
| Northeast | 0.94 | -0.69 | 2.57 | | 0.26 |
| Southeast | REFERENCE |  |  | |  |
|  |  |  |  | |  |
| Hospital Volume (mean no. of admissions per year) | | | | |  |
| ≥270 | -0.62 | -1.75 | | 0.52 | 0.29 |
| 271-470 | -0.45 | -1.61 | | 0.70 | 0.44 |
| 471-800 | -0.20 | -1.35 | | 0.96 | 0.74 |
| >800 | REFERENCE |  | |  |  |
|  |  |  | |  |  |
| Year |  |  | |  |  |
| 2011 | REFERENCE |  | |  |  |
| 2012 | -0.72 | -1.79 | | 0.35 | 0.19 |
| 2013 | -0.15 | -1.21 | | 0.91 | 0.79 |
| 2014 | -0.36 | -1.43 | | 0.70 | 0.50 |
|  |  |  | |  |  |
| Length of Stay (mean, days) | | | |  |  |
| 0-4 | REFERENCE |  | |  |  |
| 5-6 | -3.42 | -6.12 | | -0.71 | 0.01 |
| 7-10 | -2.53 | -5.09 | | 0.03 | 0.05 |
| >11 | -1.91 | -4.46 | | 0.63 | 0.14 |
|  |  |  | |  |  |
| Discharged to SNF | -0.07 | -0.82 | | 0.69 | 0.86 |
|  |  |  | |  |  |
| Constant (Days to Readmission for Reference Patient) | 14.89 | 11.88 | | 17.90 | <0.0001 |
|  |  |  | |  |  |
|  |  |  | | | |

*R-squared=0.0145. Sample Size for Matched Cohort = 1890.*
