# Supplementary material for: Readmissions attributable to skilled nursing facility use after a colectomy: Evidence using propensity scores matching
Source: PLoS One. 2019 Apr 16;14(4):e0215245. doi: 10.1371/journal.pone.0215245 (PMC6467448; doi:10.1371/journal.pone.0215245)
Supplement: S6 Table — ATT: average effect of treatment on the treated. Notes: 1) Patients were matched on all covariates included in Table 1. 2) This table shows the effect of being discharged to a SNF on readmissions rate, after the patients were matched on all covariates included in Table 1. Each patient discharged to a SNF was matched to two patients who were discharged to another destination. 3) Full set of regression results similar to those in S1 Table and S2 Table are available from the authors on request. (DOCX) [file pone.0215245.s006.docx]

S6 Table. Results of a propensity score matching analysis for 30-day readmission (discharge to SNF treated as the treatment effect; 2:1 matching)

|  | **Discharged** | **Not Discharged** |  | **95% Confidence** | |  |
| --- | --- | --- | --- | --- | --- | --- |
| **Outcome** | **to SNF** | **to SNF** | **ATT** | *Lower* | *Upper* | **P-value** |
|  |  |  |  |  |  |  |
| 30-day Readmission | 25.1% | 16.5% | 8.6% | 6.5% | 10.7% | <0.0001 |
|  |  |  |  |  |  |  |

ATT: average effect of treatment on the treated

Notes:

1) Patients were matched on all covariates included in Table 1

2) This table shows the effect of being discharged to a SNF on readmissions rate, after the patients were matched on all covariates included in Table 1. Each patient discharged to a SNF was matched to two patients who were discharged to another destination.

3) Full set of regression results similar to those in Appendix Tables A1 and A2 are available from the authors on request.
