# Supplementary material for: Readmissions attributable to skilled nursing facility use after a colectomy: Evidence using propensity scores matching
Source: PLoS One. 2019 Apr 16;14(4):e0215245. doi: 10.1371/journal.pone.0215245 (PMC6467448; doi:10.1371/journal.pone.0215245)
Supplement: S7 Table — (DOCX) [file pone.0215245.s007.docx]

S7 Table. Marginal effects from a generalized linear model regression of days to readmissions on SNF utilization on the matched cohort of patients

|  | **Marginal** | **95% Confidence** | |  |
| --- | --- | --- | --- | --- |
| **Covariate** | **Effects** | *Low* | *High* | **P-value** |
|  |  |  |  |  |
| Age (yrs) |  |  |  |  |
| 18-49 | 1.2015 | -0.8164 | 3.2195 | 0.2430 |
| 50-64 | 0.3461 | -1.0671 | 1.7592 | 0.6310 |
| 65-74 | 0.1034 | -0.8458 | 1.0526 | 0.8310 |
| ≥75 | REFERENCE |  |  |  |
|  |  |  |  |  |
| Race |  |  |  |  |
| White | REFERENCE |  |  |  |
| Black | -0.4328 | -1.6538 | 0.7882 | 0.4870 |
| Other | 0.1334 | -2.1710 | 2.4379 | 0.9100 |
|  |  |  |  |  |
| Sex |  |  |  |  |
| Male | -0.4013 | -1.1826 | 0.3799 | 0.3140 |
| Female | REFERENCE |  |  |  |
|  |  |  |  |  |
| Surgical Approach | |  |  |  |
| Laparoscopic | -0.2204 | -1.3238 | 0.8830 | 0.6950 |
| Non-Laparoscopic | REFERENCE |  |  |  |
|  |  |  |  |  |
| Primary Indication | |  |  |  |
| Diverticular Disease | REFERENCE |  |  |  |
| Cancer | -0.8861 | -2.2516 | 0.4795 | 0.2030 |
| Other | -0.9444 | -2.1358 | 0.2470 | 0.1200 |
|  |  |  |  |  |
| Ostomy |  |  |  |  |
| Yes | 0.5168 | -1.4456 | 2.4791 | 0.6060 |
| No | REFERENCE |  |  |  |
|  |  |  |  |  |
| Surgical Urgency | |  |  |  |
| Emergent | -0.2459 | -1.1396 | 0.6478 | 0.5900 |
| Urgent | 0.6582 | -0.7217 | 2.0380 | 0.3500 |
| Elective | REFERENCE |  |  |  |
|  |  |  |  |  |
| Transfer | -0.5055 | -2.4174 | 1.4065 | 0.6040 |
|  |  |  |  |  |
| Payer |  |  |  |  |
| Medicare | REFERENCE |  |  |  |
| Medicaid | 0.4754 | -1.4459 | 2.3968 | 0.6280 |
| Other Gov't Payer | -1.0443 | -6.1170 | 4.0284 | 0.6870 |
| Commercial | -0.1983 | -1.4919 | 1.0954 | 0.7640 |
| Self-Paying | 0.4312 | -5.4091 | 6.2715 | 0.8850 |
|  |  |  |  |  |
| Charlson Comorbidity Index Score | | |  |  |
| 0 | -0.1919 | -1.2737 | 0.8899 | 0.7280 |
| 1 | -0.1271 | -1.2842 | 1.0299 | 0.8290 |
| ≥2 | REFERENCE |  |  |  |
|  |  |  |  |  |
| Region of Pennsylvania | |  |  |  |
| Northwest | 0.9161 | -0.8571 | 2.6893 | 0.3110 |
| Southwest | 0.2860 | -0.6635 | 1.2355 | 0.5550 |
| North Central | 0.4999 | -1.4498 | 2.4496 | 0.6150 |
| South Central | 1.2082 | -0.2920 | 2.7084 | 0.1140 |
| Northeast | 0.9176 | -0.8387 | 2.6738 | 0.3060 |
| Southeast |  |  |  |  |
|  |  |  |  |  |
| Hospital Volume (mean no. of admissions per year) | | | |  |
| ≤270 | -0.5260 | -1.6398 | 0.5878 | 0.3550 |
| 271-470 | -0.4715 | -1.6081 | 0.6651 | 0.4160 |
| 471-800 | -0.1656 | -1.3180 | 0.9869 | 0.7780 |
| >800 | REFERENCE |  |  |  |
|  |  |  |  |  |
| Year |  |  |  |  |
| 2011 | REFERENCE |  |  |  |
| 2012 | -0.8150 | -1.8574 | 0.2275 | 0.1250 |
| 2013 | -0.1783 | -1.2372 | 0.8806 | 0.7410 |
| 2014 | -0.4083 | -1.4649 | 0.6483 | 0.4490 |
|  |  |  |  |  |
| Length of Stay (mean, days) | | |  |  |
| 0-4 | REFERENCE |  |  |  |
| 5-6 | -3.0509 | -5.2088 | -0.8931 | 0.0060 |
| 7-10 | -2.3648 | -4.7329 | 0.0032 | 0.0500 |
| >11 | -1.8676 | -4.5293 | 0.7940 | 0.1690 |
|  |  |  |  |  |
| Discharge to SNF | -0.0907 | -0.8542 | 0.6727 | 0.8160 |
|  |  |  |  |  |
